# Comparison of PLA-Based Micelles and Microspheres as Carriers of Epothilone B and Rapamycin. The Effect of Delivery System and Polymer Composition on Drug Release and Cytotoxicity against MDA-MB-231 Breast Cancer Cells

**DOI:** 10.3390/pharmaceutics13111881

**Published:** 2021-11-05

**Authors:** Katarzyna Jelonek, Alicja Zajdel, Adam Wilczok, Bożena Kaczmarczyk, Monika Musiał-Kulik, Anna Hercog, Aleksander Foryś, Małgorzata Pastusiak, Janusz Kasperczyk

**Affiliations:** 1 Centre of Polymer and Carbon Materials, Polish Academy of Sciences, 41-819 Zabrze, Poland; kjelonek@cmpw-pan.edu.pl (K.J.); bkaczmarczyk@cmpw-pan.edu.pl (B.K.); mmusial@cmpw-pan.edu.pl (M.M.-K.); ahercog@cmpw-pan.edu.pl (A.H.); aforys@cmpw-pan.edu.pl (A.F.); mpastusiak@cmpw-pan.edu.pl (M.P.); 2 Department of Biopharmacy, Faculty of Pharmaceutical Sciences in Sosnowiec, Medical University of Silesia in Katowice, 41-200 Sosnowiec, Poland; azajdel@sum.edu.pl (A.Z.); awilczok@sum.edu.pl (A.W.)

**Keywords:** microspheres, micelles, PLA, PLA–PEG, controlled drug delivery system, breast cancer, MDA-MB-231 cells, cytotoxicity, epothilone B, rapamycin

## Abstract

Co-delivery of epothilone B (EpoB) and rapamycin (Rap) increases cytotoxicity against various kinds of cancers. However, the current challenge is to develop a drug delivery system (DDS) for the simultaneous delivery and release of these two drugs. Additionally, it is important to understand the release mechanism, as well as the factors that affect drug release, in order to tailor this process. The aim of this study was to analyze PLA–PEG micelles along with several types of microspheres obtained from PLA or a mixture of PLA and PLA–PEG as carriers of EpoB and Rap for their drug release properties and cytotoxicity against breast cancer cells. The study showed that the release process of EpoB and Rap from a PLA-based injectable delivery systems depends on the type of DDS, morphology, and polymeric composition (PLA to PLA–PEG ratio). These factors also affect the biological activity of the DDS, because the cytotoxic effect of the drugs against MDA-MB-231 cells depends on the release rate. The release process from all kinds of DDS was well-characterized by the Peppas–Sahlin model and was mainly controlled by Fickian diffusion. The conducted analysis allowed also for the selection of PLA 50/PLA–PEG 50 microspheres and PLA–PEG micelles as a promising co-delivery system of EpoB and Rap.

## 1. Introduction

Drug delivery systems (DDSs) present numerous advantages by decreasing premature degradation, improving drug uptake, providing sustained drug concentrations within the therapeutic window, and reducing side effects [1]. Various carriers are developed for the release of anticancer drugs, as hydrogels or molecular imprinted polymer (MIP)-based DDSs [2,3,4,5,6]. However, although DDSs have had great success in clinics, there are still some drawbacks and limitations [7]. One of the difficulties is due to the fact that drug release from polyester-based drug delivery systems is very complex, and therefore it is important to understand the release mechanism as well as the factors that affect drug release, in order to tailor this process [8]. Polylactide (PLA) and its copolymers have a long history of safe use in humans and an extensive range of applications. PLA is biocompatible, biodegradable by hydrolysis and enzymatic activity, has a large range of mechanical and physical properties that can be engineered appropriately to suit multiple applications, and has low immunogenicity. Formulations containing PLA have also been FDA-approved for multiple applications, making it suitable for expedited clinical translatability. The rate of poly(L-lactide) degradation is very low because of its high hydrophobicity and crystalline character. The biodegradability of PLA can be tailored by copolymerization, blending, or grafting [1]. Polyethylene glycol (PEG) is the most popular hydrophilic polymer for surface modification and has been used to modify hydrophobic PLA to form amphiphilic copolymer PLA–PEG. Through copolymerization with PEG, PLA can be improved in terms of hydrophilicity, degradation rate, and crystallization, showing great potential for the development of drug carriers [9]. The amphiphilic properties of PLA–PEG are useful for the preparation of micelles by self-assembly in an aqueous solution. Polymeric micelles consist of a hydrophobic inner core, which can serve as a solubilization depot for agents with poor aqueous solubility, and a hydrophilic corona responsible for biocompatibility and prolonged biodistribution [10]. In our previous study, the biotin-functionalized PLA–PEG micelles proved to be an efficient nanocarrier for simultaneous delivery of epothilone B (EpoB) and rapamycin (Rap) to breast cancer cells [11]. Breast cancer represents the second most frequent neoplasm in humans after lung cancer. Triple-negative breast cancers (TNBC) constitute around 15% of all cases of breast cancer; globally, it is the most complex and aggressive type of breast cancer encountered in women. The TNBC is defined by its lack of expression of estrogen receptor (ER), progesterone receptor (PR), and human epidermal growth factor receptor 2 (HER2) [12,13]. Tumors with a triple negative phenotype are characterized by poor clinical prognostic features; they are usually larger in size, higher in grade, with earlier lymph node involvement, and exhibit aggressive tumor behavior, with very poor outcomes for patients [14]. In such cases, bulk chemotherapy, radiation therapy, and surgery (or their combination) are often used as treatment strategies [15]. A great deal of scientific effort has been expended on discovering therapeutic agents or a combination of drugs that provide increased cytotoxicity against TNBC. It has been determined that Rap, an inhibitor of mTOR, may be a promising agent for the treatment of breast cancer, including TNBC [16,17]. Co-administration of mTOR inhibitors with other cytostatic drugs has been considered as a strategy leading to enhanced drug efficacy. On the other hand, to develop DDS for the treatment of TNBC, e.g., nanoparticles [18,19], liposomes [20], microspheres [15,21,22,23], or micelles [24] can be applied. However, the discovery of a satisfactorily effective DDS for TNBC is still in progress. The present study is focused on an exploration of the possibility to prolong the release period of EpoB and Rap using microspheres (MS). Microspheres are defined as carriers of a monolithic spherical structure with the drug or therapeutic agent distributed throughout the polymeric matrix. MS based on biocompatible and biodegradable synthetic polymers, e.g., PLA, have been successfully studied and applied, because they can release the encapsulated drugs in a sustained manner over weeks or even for months [2]. Polymers with PEG block have been successfully used for the preparation of MS, e.g., poly(1,3-trimethylene carbonate-co-ε-caprolactone)-b-poly(ethylene glycol)-b-poly(1,3-trimethylene carbonate-co-ε-caprolactone), P(TMC-CL)_2_-PEG [25]. In the present study, the MS were obtained from PLA, PLA–PEG or a mixture of these two polymers with various ratios of PLA to PLA–PEG. The aim was to analyze the possibility of tailoring drug release time for a PLA–PEG-based DDS by the addition of a high molecular weight PLA. The encapsulation of EpoB and Rap and the drug release characteristics of the MS have been compared with PLA–PEG micelles. Moreover, the anticancer potential of the selected MS and micelles was evaluated. This is a novel approach, because PLA-based MS and PLA–PEG micelles have not been considered so far for the co-delivery of EpoB and Rap. However, even more important is the identification of factors related to the type of DDS, its polymeric composition, and morphology influencing the release kinetics of EpoB and Rap and its cytotoxicity against breast cancer cells. This outcome may be useful for the future development or improvement of biodegradable delivery systems of EpoB and/or Rap.

## 2. Materials and Methods

### 2.1. Materials

Poly(l-lactide)-co-poly(ethylene glycol) (PLA–PEG) with M_n_ of PLA = 3000 Da and M_n_ of PEG = 5000 Da (molar ratio of PEG to PLA: 71:29) was obtained from Nanosoft Polymers (Winston-Salem, NC, USA). Poly(l-lactide) with M_n_ of 115,000 Da was synthesized in the Centre of Polymer and Carbon Materials of the Polish Academy of Sciences. PVA (98–99% hydrolyzed, low molecular weight; average M.W. 11,000–31,000) was purchased from Sigma-Aldrich (Saint Louis, MO, USA). Rapamycin was purchased from LC Laboratories (Woburn, MA, USA) and epothilone B from Sigma–Aldrich. All organic solvents were of analytic grade from Sigma-Aldrich and used as received.

### 2.2. Preparation of DDS

The DDS was obtained from a single polymer (PLA or PLA–PEG) or a mixture of these two polymers according to the scheme presented in Table 1.

#### 2.2.1. Preparation of MS

The MS were prepared from PLA, PLA–PEG, or mixtures of PLA and PLA–PEG (75:25, 50:50, 25:75, 0:100 *w*/*w*%) using an oil-in-water emulsion method. Briefly, the organic phase consisted of dissolving 110 mg of polymer (drug-free MS) or 100 mg of polymer and 10 mg of drug (drug-loaded MS) in 2 mL of dichloromethane (CH_2_Cl_2_). In the case of dual drug-loaded MS, 5 mg of Rap and 5 mg of EpoB were used. The organic phase was added dropwise to the aqueous phase which consisted of 100 mL of 5% PVA and emulsified using homogenizer (Kinematica, Malters, Switzerland; Polytron PT 2500 E) at 20,200 rpm for 2.5 min. The resulting emulsion was stirred (100 rpm) overnight with a magnetic stirrer at room temperature for solvent evaporation. The obtained MS were collected by centrifugation at 12,000 rpm (Eppendorf, Hamburg, Germany; 5810R) for 15 min at 20 °C, washed three times with distilled water, lyophilized (Christ, Osterode am Harz, Germany; Alpha 1-2 LD plus) and stored at 4 °C.

#### 2.2.2. Preparation of Micelles

The PLA–PEG (PLA 0/PLA–PEG 100) was used to prepare micelles by co-solvent evaporation method [26]. Briefly, the PLA–PEG was dissolved in CH_2_Cl_2_ and mixed with deionized water at a concentration of 1 mg/mL followed by stirring at 350 rpm for 3 h at room temperature. After 24 h, the drug or mixture of drugs (EpoB/Rap; 1/1 *w*/*w*) dissolved in EtOH was added to the micelle solution (drug(s)/polymer ratio of 1/9 (*w*/*w*)) and stirred. The unloaded drug(s) was separated by centrifugation at 3000 rpm (Eppendorf 5810R) for 5 min. The micelles were lyophilized and stored at 4 °C.

### 2.3. Characterization of DDS

#### 2.3.1. Microscopic Evaluation

The morphology of the freeze-dried MS was observed using a scanning electron microscope (SEM; FEI Company, Hillsboro, OR, USA; Quanta 250 FEG). Powder samples were stuck to the microscopic stubs by the double-sided adhesive carbon type. The micrographs were obtained under low vacuum (80 Pa) with an acceleration voltage 5 kV from secondary electrons collected by a Large Field Detector (LFD). The size and size distribution of the MS was evaluated using ImageJ 1.45 s software.

The morphology of the micelles was analyzed using a transmission electron microscope (TEM; Tecnai F20 TWIN, FEI Company, Hillsboro, OR, USA) equipped with a field emission gun (200 kV). Images were recorded on the Gatan Rio 16 CMOS 4k camera (Gatan Inc., Pleasanton, CA, USA) and processed with Gatan Microscopy Suite (GMS) software (Gatan Inc., Pleasanton, CA, USA)**.** The lyophilized micelles were dissolved in deionized water (5 mg/mL) and a drop of the solution was placed on a copper grid covered with carbon film. The samples were observed after negative staining followed by air-drying at RT.

#### 2.3.2. FTIR Analysis

The potential inter- and intramolecular interactions were analyzed using Fourier Transform Infrared (FTIR) spectroscopy. The FTIR spectra were recorded with a JASCO FT/IR-6700 spectrometer (Easton, MD, USA), using a TGS detector with 64 scans and at 2 cm^−1^ resolution. The polymer matrix and the polymer matrix with the drug was analyzed as films obtained after evaporating the CHCl_3_ from their solutions on KBr windows and free drugs were analyzed as pellets in KBr.

#### 2.3.3. NMR Analysis

The free drugs, drug-free MS, and micelles, as well as particles loaded with EpoB and Rap, were analyzed by means of proton nuclear magnetic resonance (^1^H NMR) spectroscopy (Bruker, Billerica, MA, USA; Avance II Ultrashiels Plus spectrometer; 600 MHz). Before analysis, free drugs or the lyophilized DDSs (MS or micelles) were dissolved in CDCl_3_. Spectra were obtained with 32 scans, 11 ms pulse width, and 2.65 s acquisition time. Chemical shifts (δ) were given in ppm using tetramethylsilane as an internal reference.

#### 2.3.4. HPLC Measurements

The quantitative drug analysis has been conducted by means of high-performance liquid chromatography (HPLC). The measurements were conducted using a LiChrospher^®^ 100 RP-18 (5 μm) Sigma-Aldrich (Saint Louis, MO, USA), LiChroCART^®^ 250–4 column Sigma-Aldrich (Saint Louis, MO, USA) maintained at 25 °C (EpoB) or 40 °C (Rap). Analysis of EpoB was performed in 70:30 *v*/*v* of acetonitrile and water as the mobile phase with a flow rate of 0.7 mL/min. In the case of Rap, methanol and 0.1% formic acid (85:15 *v*/*v*) was used as the mobile phase and a flow rate was 1 mL/min. The volume of injection was 20 μL. EpoB was detected at 250 nm and Rap at 278 nm. The calibration curve of EpoB was linear in the studied concentration range of 0.05–100 μg/mL and *R*^2^ = 0.9997. The calibration curve of Rap was linear in the studied concentration range of 0.05–500 μg/mL and *R*^2^ = 0.9995.

### 2.4. Encapsulation Efficiency

The encapsulation efficiency (EE) was calculated using the following equation: EE = weight of the drug in the DDS/weight of the drug added to the DDS.

The determination of the drug entrapment efficiency in MS was evaluated using an extraction method [27]. Briefly, 1 mg of the MS was dissolved in 0.5 mL of methylene chloride and stirred with a magnetic stirrer at 500 rpm for 30 min, followed by drug extraction with ethanol (0.5 mL), which also promoted polymer precipitation. The samples were stirred for another 30 min, centrifuged at 5000 rpm for 15 min, and analyzed using HPLC.

For the determination of the drug entrapment efficiency in micelles, the lyophilized samples were dissolved in 0.5 mL of EtOH before quantitative assessment of the drug by means of HPLC.

### 2.5. In Vitro Release Studies

#### 2.5.1. Drug Release from MS

1 mg of the MS (Rap-loaded MS, EpoB-loaded MS, and Rap/EpoB-loaded MS) were suspended in 10 mL of phosphate buffer saline (PBS, pH 7.4) using 15 mL screw-capped tubes and incubated at 37 °C under constant agitation (100 rpm). At the specified time points (1, 6, 24, 72, 168, 336, and 720 h) the samples were centrifuged (12,000 rpm for 15 min at 20 °C), the supernatants were removed, and the precipitate (MS) was saved for analysis of the remaining drug. For this purpose, the extraction method [27] described in Section 2.3 was used. The experiment was conducted in triplicate.

#### 2.5.2. Drug Release from Micelles

The dialysis method was used for analysis of release of EpoB and Rap from micelles. The lyophilized micelles were dispersed in PBS (pH 7.4) at a concentration of 2 mg/mL. A volume of 1 mL of the micellar solution was placed in a dialysis device (Float-A-Lyzer G2, MWCO of 3.5–5 kDa; Spectra/Por). The dialysis was conducted against PBS (40 mL), which was periodically exchanged to provide sink conditions. A 25 μL of the micellar solution was collected at particular times (1, 6, 24, 72, 168, and 336 h) and replaced by fresh PBS. The samples were lyophilized and dissolved in 0.5 mL of EtOH before quantitative assessment of the drug by HPLC. The experiment was conducted in triplicate.

DDsolver, an Excel add-in program [28], was used for modeling the kinetics of the dissolution processes by fitting the dissolution profiles with time-dependent equations. Several mathematic models were applied to determine and describe drug release from the MS and micelles, e.g., Korsmeyer–Peppas and Peppas–Sahlin.

### 2.6. In Vitro Cytotoxicity Studies

To assess cytotoxic activity of free EpoB and/or Rap, the EpoB- and/or Rap-containing micelles and MS towards TNBC, a commonly used cytotoxicity sulforhodamine B based assay (TOX-6) was applied. The MDA-MB-231 cell line was purchased from ATCC. The cells were cultured in Dulbecco’s Modified Eagle’s Medium (DMEM) supplemented with 10% heat-inactivated fetal bovine serum (FBS; PAN-Biotech), 10 mM HEPES buffer (Sigma-Aldrich), 100 U/mL penicillin, and 100 μg/mL streptomycin (Sigma-Aldrich). Cells were maintained at 37 °C in a humidified atmosphere of 95% air and 5% CO_2_.

The cells seeded on the 96-well plates (1 × 10^4^ cells/well) after 24 h incubation were exposed to EpoB and Rap delivered in the tested micelles and MS for 72 h. The determination of the live cell proteins was carried out by means of sulforhodamine B assay according to the manufacturers’ protocols using the previously described procedure [25]. At the end of each test, the absorbance values that represented the live cell proteins level were measured with a plate reader (Triad LT Multimode Detector, Dynex Technologies, Chantilly, VA, USA) at 570 nm (reference wavelength 690 nm). The absorbance registered for control samples (cells not exposed to drugs or exposed to empty micelles or MS) were taken as a reference (100%) and used for comparison with those detected for the free drugs solutions and DDS that contained EpoB, Rap, or EpoB and Rap in the same concentrations. Control cells were incubated in the pure culture medium or a medium containing a non-toxic concentration of DMSO (0.1%). Where appropriate, blank micelles and MS were added in the same concentrations as in the experimental cultures.

### 2.7. Statistical Analysis

Mean values ± standard deviations were calculated from the data obtained in four independent series of experiments. Statistica 10 PL software for Windows (StatSoft, Kraków, Poland) was used to perform variance analysis (ANOVA) and Tukey’s HSD test. A *p*-value of less than 0.05 was considered significant.

## 3. Results

### 3.1. Characteristics of DDS

**Figure 1:**
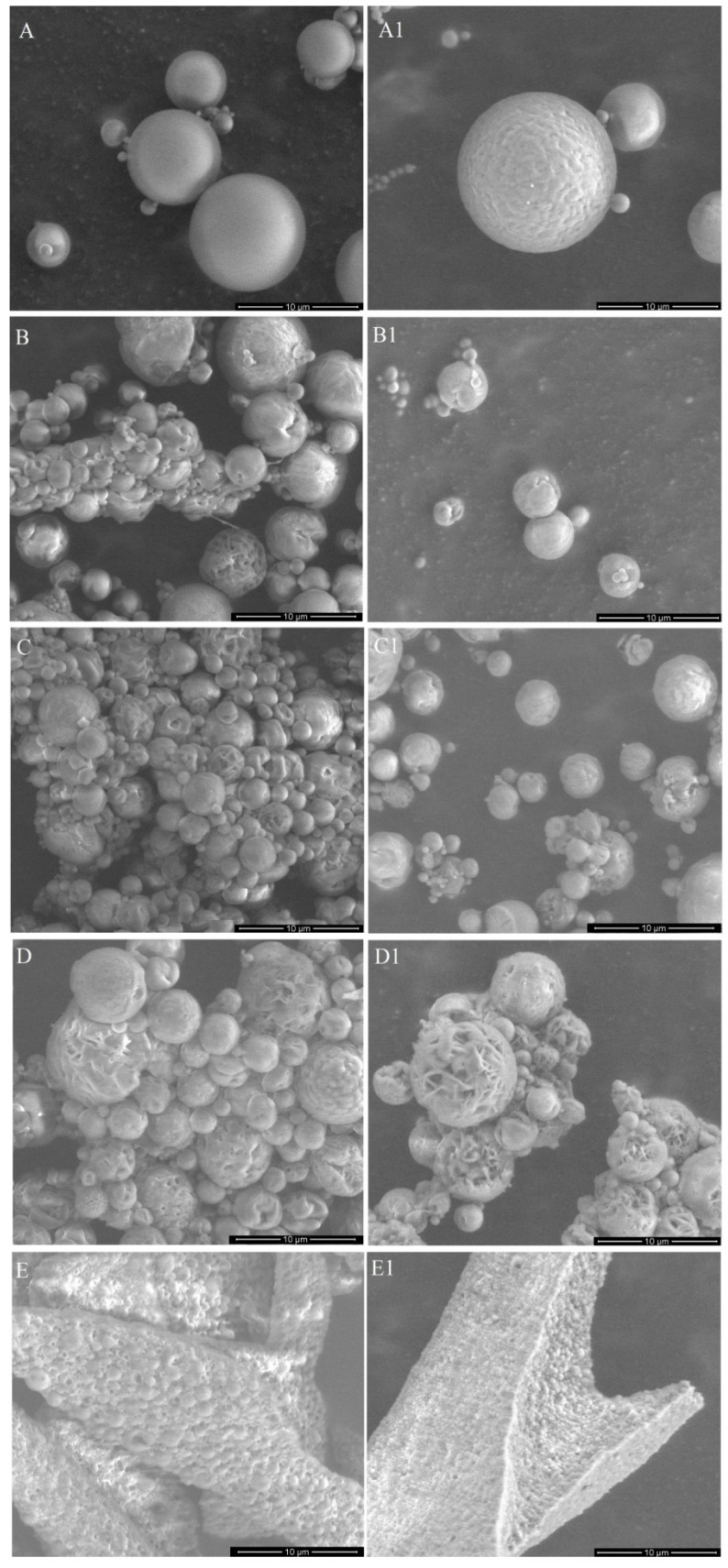
Morphology of the drug-free microspheres (MS) (**A**–**E**) and the microspheres (MS) loaded with epothilone B (EpoB) and rapamycin (Rap) (**A1**–**E1**), obtained from PLA 100/PLA–PEG 0 (**A**,**A1**), PLA 75/PLA–PEG 25 (**B**,**B1**), PLA 50/PLA–PEG 50 (**C**,**C1**), PLA 25/PLA–PEG 75 (**D**,**D1**), PLA 0/PLA–PEG 100 (**E**,**E1**).

**Figure 2:**
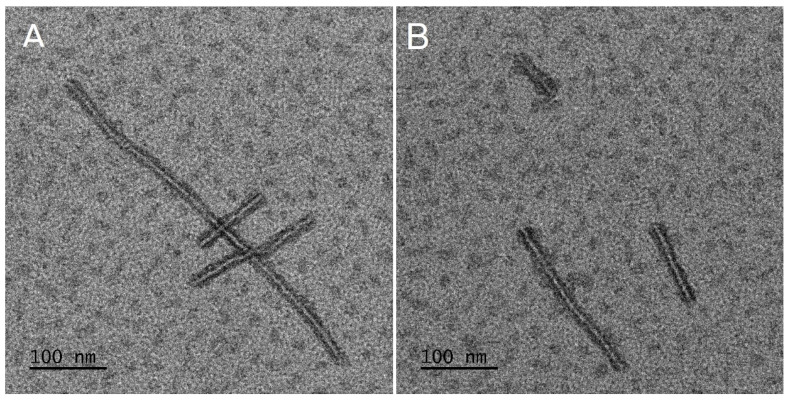
Morphology of drug-free (**A**) and epothilone B and rapamycin (EpoB + Rap) co-loaded micelles (**B**).

Two types of the DDS were obtained—MS and micelles. The MS were prepared from PLA or mixtures of PLA and PLA–PEG with various ratios of those two polymers (Table 1). Micelles were obtained from amphiphilic polymer (PLA–PEG). The morphology of the MS and micelles without the drug and co-loaded with EpoB and Rap was analyzed by means of SEM (Figure 1) or TEM (Figure 2), respectively. As shown in Figure 1, the morphology of the MS differed depending on the polymers used for their formation. PLA enabled the obtainment of a very regular spherical shape and smooth surface of the drug-free particles (Figure 1A) and the MS co-loaded with EpoB and Rap (Figure 1A1). The MS obtained from a mixture of PLA and PLA–PEG were characterized by a less compact structure—the number of microparticles with a loosened structure increased with the amount of PLA–PEG, and were in a majority in the PLA 25/PLA–PEG 75 MS (Figure 1D,D1). However, the size of all kinds of MS was similar and the average diameter of the PLA 100/PLA–PEG 0, PLA 75/PLA–PEG 25, PLA 50/PLA–PEG 50, and PLA 25/PLA–PEG 75 was 3.76 μm (±2.28 μm), 3.29 μm (±1.74 μm), 3.24 μm (±1.89 μm), and 2.47 μm (±1.22 μm), respectively (Figure 1, Figure S1). In the case of PLA 0/PLA–PEG 100, the process of MS preparation was very inefficient, and the final particles formed clusters (Figure 1E,E1). Therefore, this kind of MS was not involved in further analysis. In the case of all kinds of DDS, the morphology did not change after drug loading (Figure 1).

**Figure 3:**
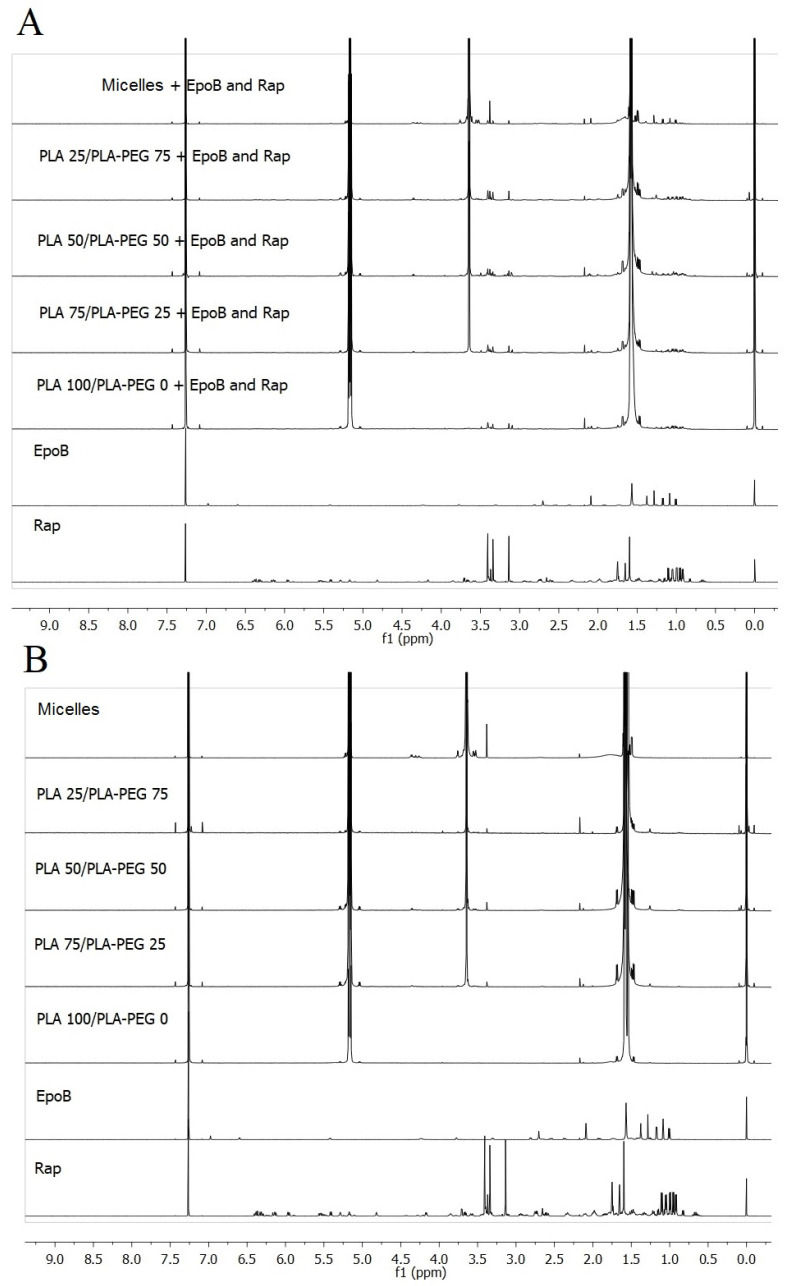
Comparison of ^1^H nuclear magnetic resonance (NMR) spectra in CDCl_3_ of free drugs and drugs-loaded delivery systems (**A**) or drug-free delivery systems (**B**).

The presence of drugs in the MS and micelles was confirmed using NMR spectroscopy. Comparison of NMR spectra of free drugs and dual drug-loaded DDSs or free drugs and drug-free DDSs is presented in Figure 3A,B, respectively. Signals assigned with the CH (5.3 ppm) and CH_3_ (1.6 ppm) groups of PLA are visible in spectra obtained for drug-free and drug-loaded DDSs. In the spectra obtained for the DDSs prepared from a mixture of PLA and PLA–PEG, CH_2_ groups of PEG are also identified at 3.6 ppm. Moreover, the signals of drugs are observed in the spectra of DDSs containing drugs. The signals assigned to CH_3_ groups of Rap are identified in all kinds of the Rap-loaded carriers at 0.95–1.11 ppm. In addition, CH_3_ groups of EpoB are visible in the spectra of EpoB-loaded DDS at 1.3 ppm.

Table 2 presents the encapsulation efficiency of the EpoB and Rap into the micelles and MS. In the case of single drug loaded DDS, the Rap encapsulation efficiency was very high (83% for PLA 100/PLA–PEG 0 + Rap and above 90% in the case of the other kinds of MS). The encapsulation properties of Rap in dual drug loaded carriers were significantly lower (from 40 to 59%). The encapsulation properties of EpoB in all kinds of MS were significantly lower and, in most cases, did not exceed 10%. Contrary to the MS, the EE of EpoB in micelles was much higher (above 40% in both single- and dual-drug loaded micelles).

### 3.2. In Vitro Drug Release

**Figure 4:**
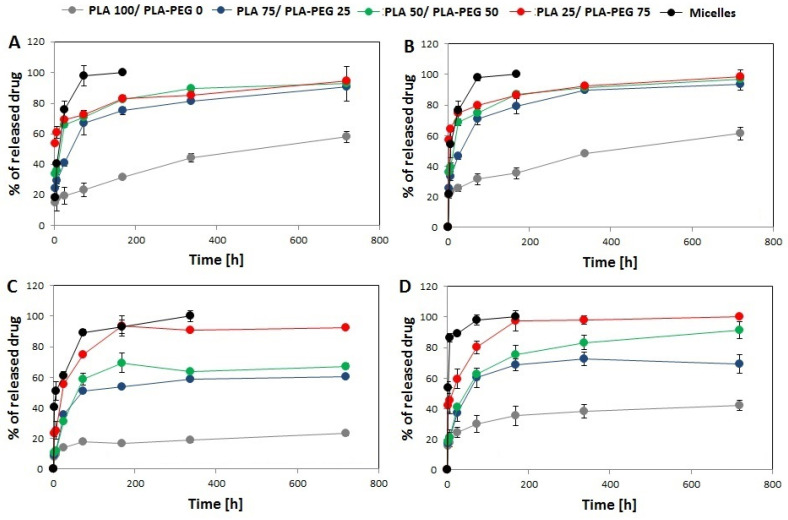
Cumulative release of rapamycine from Rap-loaded DDS (**A**), EpoB from EpoB-loaded DDS (**B**), rapamycine from Rap and EpoB-loaded DDS (**C**), and EpoB from Rap and EpoB-loaded DDS (**D**). (SD is shown as error bars, *n* = 3).

The results of in vitro drug release from the single- and dual drug-loaded DDSs are presented in the Figure 4. In all kinds of DDS, the release rate was dependent on polymer composition, so proceeded the fastest from PLA–PEG micelles (100% of drug released until 168 h) and the slowest from PLA 100/PLA–PEG 0 MS (58% of Rap and 61% of EpoB released from single drug-loaded MS after 720 h, and 23% of Rap and 42% of EpoB released from dual-drug loaded MS). The differences in drug release rate between DDSs were more significant in the case of dual drug-loaded DDSs (Figure 1C,D). The release of Rap and EpoB from single drug-loaded PLA 75/PLA–PEG 25, PLA 50/PLA–PEG 50. and PLA 25/PLA–PEG 75 proceeded with a similar rate. However, all kinds of DDS were characterized by a faster release of drugs than single drug-loaded DDS in comparison to the Rap and EpoB co-loaded DDSs.

To determine the drug release mechanism from the MS and micelles, the release data were fitted to Korsmeyer–Peppas and Peppas–Sahlin kinetic models. The comparison of the regression coefficients is presented in the Table 3. It was found that the data obtained for all kinds of the DDS were fitted well with the Peppas–Sahlin model. The Peppas–Sahlin or diffusion–relaxation model is represented by the following equation: F = *k*_1_t^m^ + *k*_2_t^2m^, where *k*_1_ and *k*_2_ are constants related to Fickian and non-Fickian kinetics, respectively, and m is the diffusional exponent for a device of any geometric shape which inhibits controlled release [26]. The detailed analysis of the parameters describing the mechanism of drug release according to the Peppas–Sahlin kinetic model is presented in the Supplementary Materials (Tables S1–S4). From Tables S1–S4, it can be observed that *k*_2_ is much smaller than *k*_1_ for all the analyzed DDSs, which means that the release from all kinds of DDS was mainly controlled by Fickian diffusion. Moreover, the increase of the *k*_1_ value correlated with the decrease of PLA.

### 3.3. FTIR Analysis

The FTIR analysis has been conducted to detect any drug–drug or polymer–drug interactions in the DDSs studied. Polymer–drug interactions or drug–drug interactions are commonly described in the literature as influencing the rate of drug diffusion and degradation kinetics [8]. Therefore, drug–polymer and drug–drug interactions are considered for the analysis of drug release mechanisms [29]. Thus, in Table 4, the assignment of the most important bands from the point of view of interactions between particular compounds (drug–drug and polymer–drug) are shown.

**Figure 5:**
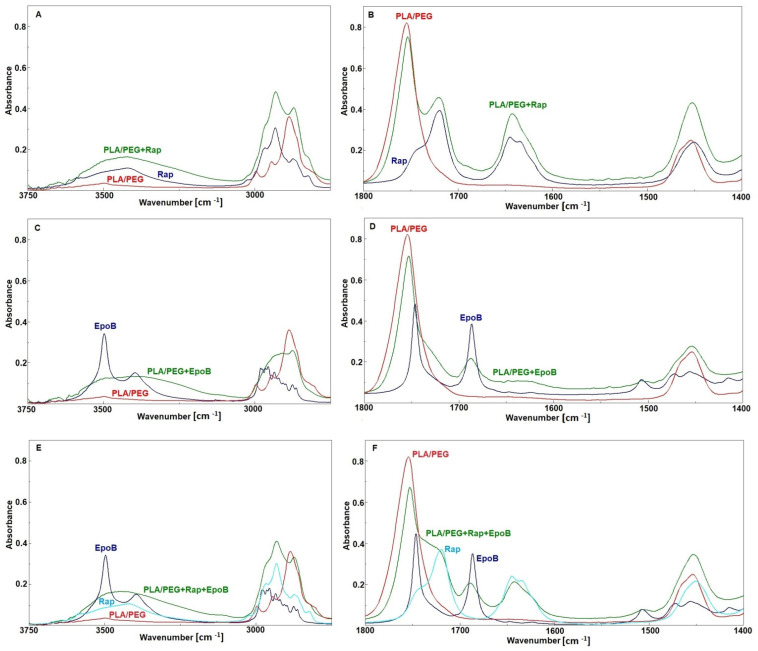
FTIR spectra of rapamycin (Rap), PLA–PEG, and PLA–PEG + Rap (**A**,**B**); epothilone B (EpoB), PLA–PEG, and PLA–PEG + EpoB (**C**,**D**); EpoB, Rap, PLA–PEG, and PLA–PEG + EpoB and Rap (**E**,**F**).

The analysis of interactions between PLA–PEG and Rap have already been described in our previous paper [30]. Similarly, in the analyzed DDSs it was demonstrated that interactions between PLA–PEG and Rap are very weak. Some small changes were observed in the region of 3500–3000 cm^−1^ (the stretching vibrations of OH groups) and for the bands at 1645 and at 1634 cm^−1^ (the stretching vibrations of the C=O ketone and the I amide band) indicate variation in hydrogen bonds distribution in Rap after mixing with a polymer matrix, and the band attributed to stretching vibration of the C=O ester group in PLA remains unchanged (Figure 5A,B). In the spectrum of EpoB, two relatively strong bands at 3497 and 3395 cm^−1^ (Figure 5C) were observed due to the stretching vibrations of free and bonded OH groups, respectively. After mixing with PLA–PEG, the broad band with many shoulders was detected in that region, suggesting that OH groups formed new hydrogen bonds. The appearance of a broad shoulder between 1740–1700 cm^−1^ (Figure 5D) indicates that these new hydrogen bonds were formed between the OH groups from EpoB and the C=O ester groups, which can derive from the polymer or the drug. Based on the FTIR spectra, as seen from the Figure 5D, this cannot be clearly determined because the bands attributed to these groups in PLA and EpoB overlap. However, the position and shape of the shoulder can indicate interactions with C=O groups of EpoB because in the spectrum of the pure drug a similar shoulder, although with a much lower intensity, is also visible. Pure EpoB exists in a crystalline form and a crystal lattice prevents the formation of the OH–O=C– hydrogen bonds due to spatial hindrance. During mixing with polymers, the crystalline form of EpoB changes and becomes amorphous and distances between OH and C=O groups decrease, which enables the formation of hydrogen bonds between EpoB molecules. Such changes in the crystallinity of drugs after mixing with polymers are known also for other drugs, e.g., for simvastatin [31]. The spatial structure of pure EpoB and changes in its structure caused by hydrogen bonds, as calculated and described in literature [32], confirms our findings.

Comparison of the spectrum obtained for the mixture of polymers with both drugs and the spectra of the pure components (Figure 5E,F) shows that the changes were similar to these observed for the mixture of PLA–PEG with EpoB. No changes were detected for the bands characteristic of Rap. This suggests that in the case of the mixture of the polymers with these two drugs the hydrogen bonds were created only between EpoB molecules and that there are no interactions between the drug and polymer matrix.

**Figure 6:**
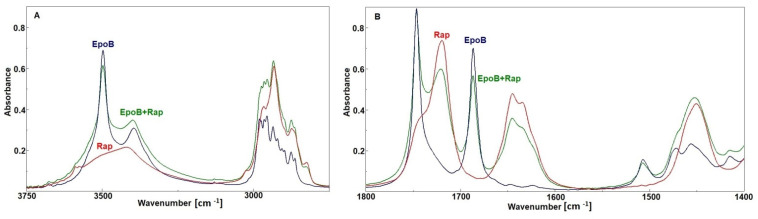
The FTIR spectra of rapamycin (Rap), epothilone B (EpoB), and a mixture of them (**A**,**B**).

In order to find out whether the analyzed drugs interact with each other, the spectrum of the Rap + EpoB mixture was recorded and compared with the spectra of particular drugs (Figure 6). It was revealed that all bands characteristic for both drugs appeared in the spectrum of the Rap and EpoB mixture, but no shifts or changes of their shapes were observed. Thus, it is proved that there is no interaction between drugs after mixing.

### 3.4. Cytotoxic Activity of EpoB and Rap

The cytotoxic effect of drugs released from micelles, or the selected MS obtained from PLA-based polymers (PLA100/PLA–PEG0 and PLA50/PLA–PEG50), was analyzed in vitro. That the live cell proteins level measured after exposure of the MDA-MB-231 cells to EpoB and Rap (Table 5) corresponded directly with the live cell number and compared with control samples that did not contain the tested drugs may reflect their toxicity. It was found that in comparison with free drugs the greatest toxic effect was achieved for the PLA–PEG micelles containing EpoB or EpoB + Rap. The live cell proteins level decreased by about 81–86%. The MS containing PLA 50/PLA–PEG 50 + EpoB and Rap inhibited cellular growth slightly less (84%); however, the difference between those two groups was not statistically significant. The mixture of free drugs at the same concentrations caused a decrease of proteins level to about 19% of the control. It should be noticed that Rap, either free or incorporated into the micelles or MS, did not exert any cytotoxic effect. Additionally, cytotoxicity was not observed in the case of PLA 100/PLA–PEG 0 MS. The results show that the observed cytotoxic effect resulted mostly from EpoB, and Rap slightly increased its toxicity.

## 4. Discussion

Drug combination is one of the strategies that can be used to overcome de novo and acquired resistance of cancer cells towards chemotherapy. It has been demonstrated that co-administration of mTOR inhibitors with other cytostatic drugs may lead to enhanced drug efficacy. The increase in cytotoxicity of a combination of EpoB and Rap against various kinds of cancers (e.g., endometrial, ovarian, non-small cell lung cancer) has been reported [33,34,35]. The enhancement of the cytotoxicity of EpoB and Rap co-loaded biodegradable biotin targeted nanocarriers against breast cancer cells was shown in our previous study [11]. However, polymeric micelles are characterized by a rather fast drug release rate. Therefore, this study is focused on assessing the possibility of extending the release period by incorporation of EpoB and Rap into the PLA-based MS. For this purpose, a series of MS with EpoB and Rap were obtained from pure PLA or from a mixture of PLA and PLA–PEG (Table 1), which were compared with PLA–PEG micelles to assess the influence of the DDS type, its morphology, and polymer composition on the drugs’ release process and cytotoxicity against breast cancer cells. The MDA-MB-231 cell line was selected for the study, because TNBC belongs to the most complex and aggressive type of breast cancer in women.

In the study, the micelles were formed from PLA–PEG and MS were prepared from five kinds of materials (PLA or a mixture of PLA and PLA–PEG) (Table 1). The MS obtained from PLA 0/PLA–PEG 100 formed clusters (Figure 1E,E1) and the process of their preparation was very inefficient, so they were excluded from further analysis. The other four kinds of MS (PLA 100/PLA–PEG 0, PLA 75/PLA–PEG 25, PLA 50/PLA–PEG 50, and PLA 25/PLA–PEG 75) were characterized by a spherical shape and the morphology did not change after drug loading (Figure 1). However, some differences in the morphology of MS were observed, because PLA formed solid particles with a smooth surface (Figure 1A,A1) and MS obtained from a mixture of PLA and PLA–PEG were characterized by a less compact structure. A correlation between the number of MS with a loosened structure and the amount of PLA–PEG was found, so they were in a majority in PLA 25/PLA–PEG 75 MS (Figure 1D,D1). Apparently, the addition of hydrophilic PLA–PEG to highly hydrophobic PLA changed the structure of the microparticles. All kinds of the MS were of a similar size, which ranged from 2.47 μm (PLA 25/PLA–PEG 75) to 3.76 μm (PLA 100/PLA–PEG 0) (Figure 1). It is worth noting that the diameter of the MS was below 10 μm, which is considered too large for phagocytosis [34]. Contrary to the round shape of the MS, PLA–PEG formed particles of an elongated shape approximately 10 nm in diameter and >200 nm in length (Figure 2).

The material used for the preparation of the DDS influenced also the in vitro drug release rate. As presented in Figure 4, the release process was polymer-dependent and proceeded fastest for PLA–PEG micelles and slowest for PLA 100/PLA–PEG 0 MS. This phenomenon may be explained by a decrease in the hydrophobicity of the material by the addition the PLA–PEG, which accelerated drug release. The second important factor related to the release process is the morphology of the DDS. More rapid drug release was observed from the core-shell micellar system. In the MS, the drug is dispersed in a solid polymer matrix, so its release is more sustained in comparison to the micelles. However, some differences are observed also for the MS, because the PLA 100/PLA–PEG 0 MS, which has the most compact structure, showed the slowest release. The highest content of PLA–PEG and the less compact structure of MS gave the fastest release of Rap and EpoB. It was found that the release data obtained for all kinds of the DDS fitted well with the Peppas–Sahlin model (Table 3) and that the release was mainly controlled by Fickian diffusion (Tables S1–S4). The Peppas–Sahlin model is used to analyze the release of pharmaceutical dosage forms, when the release mechanism is not well known or when more than one type of release phenomena could be involved and shows that drug release from any device can be considered as Fickian or non-Fickian (Case-II relaxation) transports [29,36]. The release profile of EpoB and Rap was similar, which is consistent with the FTIR analysis, which did not reveal any significant drug–drug or drug–polymer interactions that might influence the release process [30,37]. However, the formation of intramolecular interactions was observed for EpoB (Figure 5D) along with the change of its crystalline form to an amorphous state after mixing with the polymers, which may be responsible for the significantly lower encapsulation efficiency in the MS (Table 2). Interestingly, the incorporation of EpoB into the micelles proceeded with a similar effectiveness to that observed for Rap. This proves that the type of delivery system, its morphology, and preparation method strongly affects the loading properties of EpoB.

Three kinds of single and dual drug-loaded DDS have been selected for in vitro analysis of the cytotoxicity against MDA-MB-231cells: micelles, PLA 100/PLA–PEG 0 MS and PLA 50/PLA–PEG 50 MS. The micelles were included in the study for comparison of their cytotoxic effect with the MS. The selected types of the MS had various morphologies (Figure 1) and drug release rates (Figure 4), enabling the determination of the influence of these factors on the proliferation of MDA-MB-231 cells. The decrease of cell proliferation was observed only in the presence of micelles or PLA 50/PLA–PEG 50 MS loaded with EpoB or EpoB and Rap. The greatest toxic effect was achieved for micelles containing EpoB or EpoB + Rap, which showed the fastest release process. The drugs-loaded PLA 100/PLA–PEG 0 MS did not affect cell growth, which was probably due to the slow release process and an insufficient drug dose. As presented in Figure 4, the quantity of drugs released from PLA 100/PLA–PEG 0 MS was significantly lower in comparison to the other kinds of DDS. Thus, the drug dose released from PLA 100/PLA–PEG 0 MS after 72 h was apparently too low to exhibit a cytotoxic effect.

In addition, the Rap-loaded DDS did not cause any cytotoxic effect, which is consistent with our previous study [11]. Importantly, all the drug-free DDSs did not affect cell proliferation, which proves their cytocompatibility.

## 5. Conclusions

In this study, a series of microspheres with EpoB and Rap were obtained from pure PLA or from a mixture of PLA and PLA–PEG. The MS were compared with PLA–PEG micelles to assess the influence of DDS type, morphology, and polymer composition on the drugs’ release process and on cytotoxicity against breast cancer cells. All kinds of MS had a spherical shape and a similar size (approximately 3 µm) but different morphologies—more solid in the case of MS obtained from pure PLA (PLA 100/PLA–PEG 0 MS), while the structure loosened with the increase of PLA–PEG content. Drug incorporation into micelles and microspheres was confirmed by means of NMR spectroscopy. Comparison of the FTIR spectra of polymers and polymer–drug mixtures revealed that there was no significant modification of the chemical groups of polymers due to drug loading. However, the intramolecular interactions of epothilone B and the change of its crystalline form to an amorphous state after mixing with polymers may be responsible for significantly lower encapsulation efficiency in the MS. Higher encapsulation efficiency for EpoB in the micelles proves that the drug loading process depends also on the type of delivery system. The study revealed also that the in vitro release process of drugs from single and dual drug-loaded DDSs depends on polymer composition, so it was slowest from PLA 100/PLA–PEG 0 MS and the release rate increased with the addition of PLA–PEG. The fastest drug release process was observed for the PLA–PEG micelles. It was found that the release data obtained for all kinds of DDS fitted well with the Peppas–Sahlin model and that the release was mainly controlled by Fickian diffusion. The biological activity of the developed PLA-based micelles and MS was studied in vitro on MDA-MB-231 cells. The decrease of cell growth was observed in the presence of micelles or PLA 50/PLA–PEG 50 MS loaded with EpoB or EpoB and Rap. The drugs loaded with PLA 100/PLA–PEG 0 MS showed no toxic effect, which was probably caused by the drug release being too slow. Importantly, all the drug-free DDSs did not affect cell growth, which proves their cytocompatibility.

The conducted analysis allowed the identification of PLA 50/PLA–PEG 50 microspheres and PLA–PEG micelles as a promising co-delivery system of epothilone B and rapamycin. These DDSs showed a high drug encapsulation efficiency and optimal release rate and provided a dose sufficient to exert a cytotoxic effect on cancer cells. The study also shows that the release process of epothilone B and rapamycin from PLA-based injectable delivery systems depends on the type of DDS, morphology, and polymeric composition. These factors also affect the biological activity of the DDS, because the cytotoxic effect of the drugs depends on the release rate.

## Figures and Tables

**Table 1:** The polymeric composition of the studied delivery systems (DDS) of epothilone B (EpoB) and rapamycin (Rap).

Name of DDS	Composition of DDS	
	Amount of PLA (%)	Amount of PLA–PEG (%)
Microspheres		
PLA 100/PLA–PEG 0	100	0
PLA 75/PLA–PEG 25	75	25
PLA 50/PLA–PEG 50	50	50
PLA 25/PLA–PEG 75	25	75
PLA 0/PLA–PEG 100	0	100
Micelles		
Micelles	0	100

**Table 2:** Comparison of the drug encapsulation properties of epothilone B (EpoB) and rapamycin (Rap) by various PLA-based delivery systems (DDS).

Name of DDS	EE of EpoB (%)	EE of Rap (%)
PLA 100/PLA–PEG 0 + EpoB	8.9 ± 1.2	-
PLA 100/PLA–PEG 0 + Rap	-	83.0 ± 4.2
PLA 100/PLA–PEG 0 + EpoB and Rap	13.1 ± 2.3	48.1 ± 1.5
PLA 75/PLA–PEG 25 + EpoB	7.2 ± 2.3	-
PLA 75/PLA–PEG 25 + Rap	-	96.0 ± 3.7
PLA 75/PLA–PEG 25 + EpoB and Rap	9.4 ± 1.9	54.2 ± 0.9
PLA 50/PLA–PEG 50 + EpoB	2.9 ± 0.2	
PLA 50/PLA–PEG 50 + Rap	-	95.0 ± 4.7
PLA 50/PLA–PEG 50 + EpoB and Rap	4.2 ± 1.2	59.7 ± 1.3
PLA 25/PLA–PEG 75 + EpoB	6.8 ± 3.6	-
PLA 25/PLA–PEG 75 + Rap	-	91.0 ± 8.9
PLA 25/PLA–PEG 75 + EpoB and Rap	7.2 ± 2.7	53.9 ± 1.8
Micelles + EpoB	43.8 ± 3.8	
Micelles + Rap	-	96.5 ± 3.3
Micelles + EpoB and Rap	41.0 ± 3.8	40.3 ± 0.7

**Table 3:** Comparison of the regression coefficients (*R*^2^) obtained after fitting the drug release data to the Korsmeyer–Peppas and Peppas–Sahlin model.

Name of DDS	Korsmeyer-Peppas (*R*^2^)		Peppas-Sahlin (*R*^2^)	
	EpoB	Rap	EpoB	Rap
PLA 100/PLA–PEG 0 + EpoB	0.945	-	0.952	-
PLA 100/PLA–PEG 0 + Rap	-	0.951	-	0.960
PLA 100/PLA–PEG 0 + EpoB and Rap	0.995	0.975	0.995	0.975
PLA 75/PLA–PEG 25 + EpoB	0.977	-	0.992	-
PLA 75/PLA–PEG 25 + Rap	-	0.976	-	0.985
PLA 75/PLA–PEG 25 + EpoB and Rap	0.924	0.909	0.988	0.971
PLA 50/PLA–PEG 50 + EpoB	0.972	-	0.985	-
PLA 50/PLA–PEG 50 + Rap	-	0.969	-	0.982
PLA 50/PLA–PEG 50 + EpoB and Rap	0.972	0.876	0.992	0.961
PLA 25/PLA–PEG 75 + EpoB	0.999	-	0.999	-
PLA 25/PLA–PEG 75 + Rap	-	0.996	-	0.997
PLA 25/PLA–PEG 75 + EpoB and Rap	0.968	0.925	0.977	0.982
Micelles + EpoB	0.956	-	0.996	-
Micelles + Rap	-	0.919	-	0.988
Micelles + EpoB and Rap	0.966	0.982	0.976	0.982

**Table 4:** Assignment of the bands in selected regions of FTIR spectra for the investigated compounds.

PLA–PEG	Rap	EpoB	Assignment
3500	3588 3577 sh 3419	3497 3395	Overtonevν OH
3000–2700	3000–2700	3000–2700	ν CH, CH_2_
1754			ν C=O ester PLA
	1720 1645 1634	1746 1742 sh 1686	ν C=O ester free ν C=O ester bonded ν C=O ketone ν C=O amide (I amide bands)

ν—stretching vibrations.

**Table 5:** Live cell proteins level expressed as a percent of controls (mean values ± standard deviations) in MDA-MB-231 cells after 72 h exposure with free EpoB and Rap, and the drugs-loaded micelles and microspheres measured with sulforhodamine B assay (TOX-6). Statistically significant differences vs. controls were denoted by asterisks (*p* < 0.05).

Drugs Concentration	Free Drugs	Micelles PLA100/PLA–PEG0	Microspheres PLA100/PLA–PEG0	Microspheres PLA50/PLA–PEG50
Control (blank)	100.00 ± 0.44	100.00 ± 0.29	100.00 ± 0.45	100.00 ± 0.24
EpoB 10 nM	24.67 ± 1.11 *	18.77 ± 1.23 *	100.25 ± 0.54	19.58 ± 1.32 *
Rap 4 nM	99.22 ± 1.42	98.89 ± 1.72	98.42 ± 0.51	97.63 ± 1.22
EpoB 10 nM + Rap 4 nM	19.52 ± 1.23 *	14.18 ± 1.75 *	97.29 ± 0.97	16.25 ± 1.39 *

## Data Availability

Not applicable.

## Supplementary Materials

The following are available online at ’https://www.mdpi.com/article/10.3390/pharmaceutics13111881/s1. Table S1: The parameters of the Peppas–Sahlin model demonstrated for the single drug-loaded DDS with epothilone B (EpoB); Table S2: The parameters of the Peppas–Sahlin model demonstrated for the dual drug-loaded DDS with epothilone B (EpoB); Table S3: The parameters of the Peppas–Sahlin model demonstrated for the single drug-loaded DDS with rapamycin (Rap); Table S4: The parameters of the Peppas-Sahlin model demonstrated for the dual drug-loaded DDS with rapamycin (Rap); Figure S1: Histogram presenting distribution of dimensions of the microspheres; Figure S2. Histogram presenting distribution of dimensions of the micelles.
