# Supplementary material for: Comparison of PLA-Based Micelles and Microspheres as Carriers of Epothilone B and Rapamycin. The Effect of Delivery System and Polymer Composition on Drug Release and Cytotoxicity against MDA-MB-231 Breast Cancer Cells
Source: Pharmaceutics. 2021 Nov 5;13(11):1881. doi: 10.3390/pharmaceutics13111881 (PMC8624627; doi:10.3390/pharmaceutics13111881)
Supplement: Supplementary file 1 [file pharmaceutics-13-01881-s001.zip › pharmaceutics-1420415 supp revised.pdf]

pharmaceutics

Supplementary Materials: Comparison of PLA-Based Micelles and Microspheres as Carriers of Epothilone B and Rapamycin. The Effect of Delivery System and Polymer Composition on Drug Release and Cytotoxicity against MDA-MB-231 Breast Cancer Cells

Katarzyna Jelonek, Alicja Zajdel, Adam Wilczok, Bożena Kaczmarczyk, Monika Musiał-Kulik, Anna Hercog, Aleksander Foryś, Małgorzata Pastusiak and Janusz Kasperczyk

Table S1. The parameters of Peppas-Sahlin model demonstrated for the single-drug loaded DDS with epothilone B (EpoB).

Name of DDS	Peppas-Sahlin			
	**(R^2)**	k_1	k_2	m
PLA 100/ PLA-PEG 0 + EpoB	0.952	11.629	3.205	0.155
PLA 75/ PLA-PEG 25 + EpoB	0.992	22.307	−1.323	0.320
PLA 50/ PLA-PEG 50 + EpoB	0.985	35.609	−3.283	0.251
PLA 25/ PLA-PEG 75 + EpoB	0.999	55.800	0.644	0.083
Micelles + EpoB	0.996	27.520	−1.860	0.422

Table S2. The parameters of Peppas-Sahlin model demonstrated for the dual-drug loaded DDS with epothilone B (EpoB).

Name of DDS	Peppas-Sahlin			
	**(R^2)**	k_1	k_2	m
PLA 100/ PLA-PEG 0 + EpoB and Rap	0.995	14.749	0.034	0.163
PLA 75/ PLA-PEG 25 + EpoB and Rap	0.988	13.302	−0.593	0.405
PLA 50/ PLA-PEG 50 + EpoB and Rap	0.992	14.302	−0.562	0.388
PLA 25/ PLA-PEG 75 + EpoB and Rap	0.977	36.996	−3.359	0.255
Micelles + EpoB	0.996	27.520	−1.860	0.422

Table S3. The parameters of Peppas-Sahlin model demonstrated for the single-drug loaded DDS with rapamycin (Rap).

Name of DDS	Peppas-Sahlin			
	**(R^2)**	k_1	k_2	m
PLA 100/ PLA-PEG 0 + Rap	0.960	7.356	1.624	0.211
PLA 75/ PLA-PEG 25 + Rap	0.985	20.324	−1.123	0.316
PLA 50/ PLA-PEG 50 + Rap	0.982	32.320	−2.802	0.263
PLA 25/ PLA-PEG 75 + Rap	0.997	37.629	14.863	0.066
Micelles + Rap	0.988	23.279	−1.277	0.423

Table S4. The parameters of Peppas-Sahlin model demonstrated for the dual-drug loaded DDS with rapamycin (Rap).

Name of DDS	Peppas-Sahlin			
	**(R^2)**	k_1	k_2	m
PLA 100/ PLA-PEG 0 + EpoB and Rap	0.975	8.063	0.035	0.155
PLA 75/ PLA-PEG 25 + EpoB and Rap	0.971	9.804	−0.383	0.421
PLA 50/ PLA-PEG 50 + EpoB and Rap	0.961	9.029	−0.280	0.466
PLA 25/ PLA-PEG 75 + EpoB and Rap	0.982	18.656	−0.895	0.390
Micelles + EpoB and Rap	0.982	20.125	−1.108	0.418

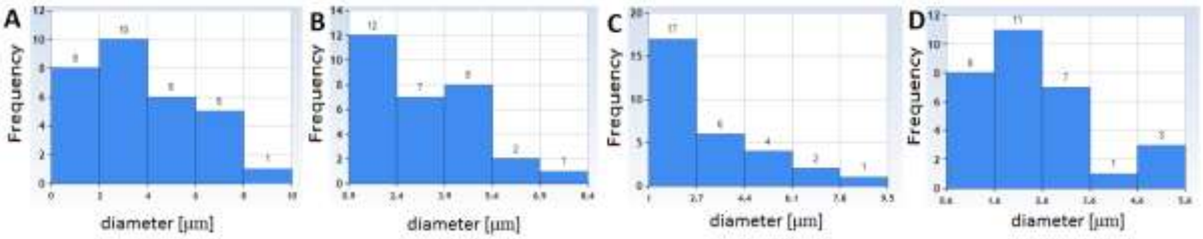

Figure S1. Histogram presenting distribution of dimensions of the microspheres obtained from (**A**) PLA 100/PLA–PEG 0; (**B**) PLA 75/PLA–PEG 25; (**C**) PLA 50/PLA–PEG 50; (**D**) PLA 25/PLA–PEG 75.

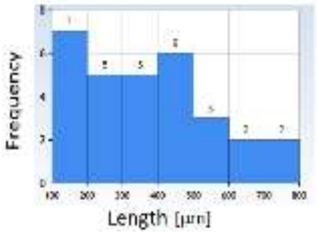

Figure S2. Histogram presenting distribution of dimensions of the micelles.
